# Exploring the Association between Mediterranean Diet Adherence and Arterial Stiffness in Healthy Adults: Findings from the EvasCu Study

**DOI:** 10.3390/nu16132158

**Published:** 2024-07-06

**Authors:** Iris Otero-Luis, Alicia Saz-Lara, Nerea Moreno-Herráiz, Carla Geovanna Lever-Megina, Bruno Bizzozero-Peroni, Isabel Antonia Martínez-Ortega, Rebeca Varga-Cirila, Iván Cavero-Redondo

**Affiliations:** 1 CarVasCare Research Group (2023-GRIN-34459), Facultad de Enfermería de Cuenca, Universidad de Castilla-La Mancha, 16002 Cuenca, Spain; iris.otero@uclm.es (I.O.-L.); nerea.moreno@uclm.es (N.M.-H.); carlageovanna.lever@alu.uclm.es (C.G.L.-M.); rebecaelenavictoria.varga@alu.uclm.es (R.V.-C.); ivan.cavero@uclm.es (I.C.-R.); 2 Instituto Superior de Educación Física, Universidad de la República, Rivera 40000, Uruguay; bruno.bizzozero@uclm.es; 3 Health and Social Research Center, Universidad de Castilla La-Mancha, 16002 Cuenca, Spain; isabela.martinez@uclm.es; 4 Facultad de Ciencias de la Salud, Universidad Autónoma de Chile, Talca 3460000, Chile

**Keywords:** Mediterranean diet, MEDAS-14 questionnaire, arterial stiffness, pulse wave velocity, healthy adults

## Abstract

(1) Background: Previous evidence has indicated a connection between a Mediterranean diet and cardiovascular disease. However, evidence for subclinical markers of cardiovascular disease, such as arterial stiffness, is limited. Therefore, the aim of this study was to assess the associations between adherence to the Mediterranean diet (MD), as assessed by the MEDAS-14 questionnaire, and arterial stiffness, as assessed by aortic pulse wave velocity, in healthy adults and according to sex. (2) A cross-sectional study including 386 healthy participants was performed in the EVasCu study. Adjusted and unadjusted differences in adherence to the MD and arterial stiffness were determined using Student’s *t* test and ANCOVA for the total sample and according to sex. (3) Results: Our results showed that individuals with a high adherence to the MD had a greater arterial stiffness, both in the total sample and in females, although this difference was not significant after adjusting for possible confounding variables, such as age. (4) Conclusions: Our findings indicated that, in the unadjusted analyses, healthy subjects with a high adherence to the MD showed a greater arterial stiffness. When these analyses were adjusted, no significant differences were shown in a-PWv according to the categories of MD adherence.

## 1. Introduction

Arterial stiffness is directly associated with cardiovascular disease (CVD), which is the leading cause of death in both women and men [1,2] and is a major risk factor for global morbidity [2]. Aortic pulse wave velocity (a-PWV) is a direct measure of the stiffness of large arteries [1]. Increased arterial stiffness can lead to elevated blood pressure and other dysfunctions, such as left ventricular hypertrophy, coronary ischemic disease, decreased ventricular diastolic function, and a reduced sensitivity of the arterial baroreceptors [2]. Furthermore, arterial stiffness is recognized as a direct predictor of cardiovascular mortality, as it is associated with an increased risk of these cardiovascular events [2,3].

The Mediterranean diet (MD) is widely accepted as a pattern of eating that promotes health in countries in the Mediterranean region [4]. This type of diet is characterized by the promotion of a high intake of monounsaturated fats, mainly obtained from olive oil, as well as fruits, vegetables, nuts, legumes, and cereals as the main fat and fiber sources. In addition, it advocates a moderate intake of dairy products, fish, poultry, and wine [4,5], while limiting red and processed meat consumption and sweets (e.g., carbonated beverages and pastries) [4]. In summary, this type of diet is characterized by a low intake of saturated fats and provides essential vitamins and minerals due to an increased consumption of fruits, vegetables, and olive oil [4]. Cardiovascular events and cardiovascular mortality are significantly reduced when associated with the MD [6] in both healthy and unhealthy populations [7].

According to the available evidence, if the MD is tailored to meet the dietary recommendations for adults, it is effective in improving cardiovascular health, significantly reducing arterial stiffness and variability in cardiovascular response in both men and women [8]. A study conducted on patients with cardiovascular risk factors, such as obesity, suggested that, to prevent arterial stiffness, a diet similar to the MD should be followed, in that it should be rich in dairy products, vegetable oils, and fish, with a reduced consumption of red meat and five daily servings of fruits and vegetables [9]. Another cross-sectional study suggested that healthy habits based on the MD combined with regular physical activity were associated with a lower arterial stiffness in a healthy population over 65 years of age who were overweight or obese [10].

Although previous evidence on the association between the MD and CVD exists [6,7,8,9,10], evidence supporting an association between the MD and arterial stiffness, considered as a subclinical marker of CVD, is limited. Therefore, the aims of this study were (i) to evaluate the association between adherence to the MD, as assessed by the MEDAS-14 questionnaire, and arterial stiffness, as assessed by a-PWV, in healthy adults; (ii) to assess the association of adherence to the MD and its specific items with arterial stiffness according to sex; and (iii) to evaluate the association between adherence to MD items and arterial stiffness in the total sample and according to sex.

## 2. Materials and Methods

### 2.1. Design, Participants, and Sample Size

The cross-sectional EVasCu study used data from healthy adults in the city of Cuenca (Spain) to assess the validity of an early vascular aging model as a cardiovascular risk index. Healthy subjects (over 18 years old) who were clinically stable during the 6 weeks prior to the study and had previously given written consent were included in the study. Patients were excluded if they were participating in another study; had a diagnosis of pathology (diabetes mellitus, arterial hypertension, cancer, acute myocardial infarction, angina pectoris, chronic kidney disease, chronic obstructive pulmonary disease, or hypercholesterolemia), whose data were collected via a self-reported questionnaire; or were receiving pharmacological treatment. A total of 406 participants were recruited through community announcements (information posters). Of these, 16 were excluded because they did not meet the inclusion criteria; therefore, 390 participants were ultimately included in our study. The people who participated in the EVasCu study were enrolled from June to December 2022. This study adhered to the Strengthening the Reporting of Observational Studies in Epidemiology (STROBE) statement [11].

### 2.2. Ethical Considerations

The EVasCu study was previously approved by the Clinical Research Ethics Committee of the Cuenca Health Area (REG: 2022/PI2022). In addition, written informed consent was obtained from all the subjects included in the study.

### 2.3. Variables

Arterial stiffness was assessed by oscillometric techniques using Mobil-o-Graph^®^ (IEM GmbH, Stolberg, Germany), which measures a-PWV. The a-PWV was calculated by averaging two repeated measurements, with a 5-min interval between each. Measurements were performed in a quiet environment following a 5-min rest period utilizing cuffs sized according to the participant’s arm circumference. Assessments of arterial stiffness have been performed by measuring a-PWV, as there is an association between an increased arterial stiffness and elevated a-PWV [12].

Adherence to the MD was assessed using the self-reported MEDAS-14 questionnaire, which consists of 14 items [13]. MD adherence was categorized as low when the score was equal to or less than 7, medium when the score was between 8 and 9, and high when the score was equal to or greater than 10 [14]. When the questionnaire items were used, adherence to the MD was categorized as adherence or nonadherence.

### 2.4. Covariates

The covariates included sex, age, body mass index (BMI), educational level, and pulse pressure (PP). Gender and educational level were obtained through direct self-reported questions. For gender, the possible answers were male or female, and for educational level, the options were cannot read or write/no education, primary education, secondary education/vocational training/higher education, or university degree. BMI was calculated using the following formula: [weight (kg)/height (m^2^)]. PP was obtained from the difference between the mean SBP and mean DBP. Blood pressure was quantified in a quiet environment following a 5-min rest period using an Omron^®^ M5-I monitor (Omron Healthcare UK Ltd., Milton Keynes, UK) with a cuff size appropriate for the participant’s arm circumference. SBP and DBP were calculated as the means of two repeated measurements, with 5-min intervals between each measurement.

Sex, BMI [15,16], physical status [17,18], and educational level [15,19] were analyzed as potential confounding factors, as differences in these variables may affect both adherence to the MD and a-PWV [20,21].

### 2.5. Statistical Analysis

Normal probability plots and the Kolmogorov-Smirnov test were used to assess the normality of the distribution of the continuous variables, where a *p* value > 0.05 indicated normality of the continuous variables. Descriptive data for the total sample are presented as means and standard deviations (SDs) or proportions (%), where appropriate. Student’s t test for independent samples and analysis of covariance (ANCOVA) were performed for the total sample between arterial stiffness and adherence to the MD (low, medium, or high). In addition, ANCOVA was performed adjusting for all variables, including age (model 1), educational level (cannot read or write/no education, primary education, secondary education/vocational training/higher education, or university degree), BMI, and PP (model 2). All analyses were performed by sex (males or females). Furthermore, all analyses were performed for each MEDAS-14 questionnaire item for the total sample and by gender, where adherence to the MD was categorized as adherence or nonadherence.

All the statistical analyses were conducted using IBM SPSS 28 (SPSS Inc., Chicago, IL, USA), and a significance level of *p* < 0.05 was considered to indicate statistical significance.

## 3. Results

### 3.1. Sample Characteristics

From the total sample of the EVasCu study, 386 healthy subjects were included (4 subjects were excluded because of missing a-PWV data), of whom 143 were males and 243 were females. The mean age of the participants was 41.92 ± 13.14 years. The baseline characteristics of the participants are shown in Table 1.

### 3.2. Association between Arterial Stiffness and Adherence to the Mediterranean Diet

Table 2 displays the unadjusted and adjusted differences in arterial stiffness according to MD adherence (low, medium, or high). For the total sample, in the unadjusted analyses, patients with a low adherence had lower a-PWV values than those with a high adherence to the MD did (5.91 ± 1.33 m/s vs. 6.63 ± 1.27 m/s) (unadjusted model, *p* < 0.001). When analyses were adjusted, no significant differences were shown in a-PWV according to the categories of DM adherence (model 1, *p* = 0.843 and model 2, *p* = 0.695). According to the analyses stratified by sex, for females, in the unadjusted analyses, patients with a low adherence had lower a-PWV values than those with a high adherence to the MD did (5.72 ± 1.29 m/s vs. 6.60 ± 1.29 m/s) (unadjusted model, *p* < 0.001). When analyses were adjusted, no significant differences were shown in a-PWV according to the categories of DM adherence (model 1 *p* = 0.831 and model 2, *p* = 0.566). For males, no significant differences were detected regarding the association between adherence to the MD and arterial stiffness.

### 3.3. Association between Arterial Stiffness and Adherence to the Mediterranean Diet According to the MEDAS-14 Questionnaire Items

Table 3 shows the unadjusted and adjusted differences in arterial stiffness according to MD adherence (adherence or nonadherence) for each item of the MEDAS-14 questionnaire. For item 2 (daily olive oil intake), adherence to the MD (≥4 tablespoons) had greater a-PWV than nonadherence did (6.49 ± 1.35 m/s vs. 6.03 ± 1.30 m/s) (unadjusted model, *p* = 0.002; model 1 *p* = 0.898 and model 2, *p* = 0.694). For item 3 (daily vegetable intake), adherence to the MD (≥2 servings) had greater a-PWV than nonadherence did (6.49 ± 1.36 m/s vs. 6.00 ± 1.28 m/s) (unadjusted model, *p* < 0.001; model 1 *p* = 0.756 and model 2, *p* = 0.918). For item 4 (daily fruit intake), adherence to the MD (three or more pieces per day) had greater a-PWV than nonadherence did (6.64 ± 1.28 m/s vs. 6.07 ± 1.36 m/s) (unadjusted model, *p* < 0.001; model 1 *p* = 0.248 and model 2, *p* = 0.285). For item 8 (weekly wine consumption), adherence to the MD (seven or more glasses per week) had greater a-PWV values than nonadherence did (7.33 ± 1.33 m/s vs. 6.26 ± 1.32 m/s) (unadjusted model, *p* < 0.001; model 1 *p* = 0.601 and model 2, *p* = 0.657). For item 10 (weekly fish/seafood servings), adherence to the MD (three or more servings per week) had greater a-PWV values than nonadherence did (6.51 ± 1.40 m/s vs. 6.14 ± 1.27 m/s) (unadjusted model, *p* = 0.007; model 1 *p* = 0.171 and model 2, *p* = 0.184). Finally, for item 12 (weekly nut consumption), adherence to the MD (three or more servings per week) had greater a-PWV values than nonadherence did (6.46 ± 1.40 m/s vs. 6.11 ± 1.23 m/s) (unadjusted model, *p* = 0.015; model 1 *p* = 0.651 and model 2, *p* = 0.933).

Table 4 shows the unadjusted and adjusted differences in arterial stiffness according to MD adherence (adherence and nonadherence) for each item of the MEDAS-14 questionnaire for males. For item 8 (weekly wine consumption), adherence to the MD (seven or more glasses per week) had greater a-PWV values than nonadherence did (7.57 ± 1.54 m/s vs. 6.38 ± 1.25 m/s) (unadjusted model, *p* < 0.001; model 1 *p* = 0.293 and model 2, *p* = 0.443). For item 9 (weekly legume consumption), adherence to the MD (three or more servings per week) had greater a-PWV values than nonadherence did (6.68 ± 1.46 m/s vs. 6.33 ± 1.15 m/s) (unadjusted model, *p* = 0.117; model 1 *p* = 0.036 and model 2, *p* = 0.024).

Table 5 shows the unadjusted and adjusted differences between arterial stiffness and adherence to the MD (adherence and nonadherence) for each item of the MEDAS-14 questionnaire for females. For item 2 (daily olive oil intake), adherence to the MD (≥4 tablespoons) had greater a-PWV values than nonadherence did (6.41 ± 1.36 m/s vs. 5.74 ± 1.20 m/s) (unadjusted model, *p* < 0.001; model 1 *p* = 0.953 and model 2, *p* = 0.812). For item 3 (daily vegetable intake), adherence to the MD (≥2 servings) had greater a-PWV than nonadherence did (6.42 ± 1.35 m/s vs. 5.70 ± 1.22 m/s) (unadjusted model, *p* < 0.001; model 1 *p* = 0.460 and model 2, *p* = 0.966). For item 4 (daily fruit intake), adherence to the MD (three or more pieces per day) had greater a-PWV values than nonadherence did (6.69 ± 1.28 m/s vs. 5.85 ± 1.29 m/s) (unadjusted model, *p* < 0.001; model 1 *p* = 0.411 and model 2, *p* = 0.568). For item 10 (weekly fish/seafood servings), adherence to the MD (three or more servings per week) had greater a-PWV values than nonadherence did (6.43 ± 1.42 m/s vs. 6.00 ± 1.24 m/s) (unadjusted model, *p* = 0.012; model 1 *p* = 0.274 and model 2, *p* = 0.267). For item 12 (weekly nut consumption), adherence to the MD (three or more servings per week) had greater a-PWV values than nonadherence did (6.38 ± 1.43 m/s vs. 5.96 ± 1.17 m/s) (unadjusted model, *p* = 0.018; model 1 *p* = 0.829 and model 2, *p* = 0.329). For item 14 (consumption of dressings such as tomato sauce, garlic, onion, or leek cooked slowly with olive oil), adherence to the MD (two or more servings per week) showed greater a-PWV than nonadherence (6.25 ± 1.31 m/s vs. 6.13 ± 1.53 m/s) (unadjusted model, *p* = 0.577; model 1 *p* = 0.034 and model 2, *p* = 0.004).

## 4. Discussion

The aim of this study was to evaluate the association between adherence to the MD, as assessed by the MEDAS-14 questionnaire, and arterial stiffness, as assessed by a-PWV, in healthy adults and according to sex. Our results indicated, in the unadjusted analyses, that individuals with a high adherence to the MD had higher a-PWV, both in the total sample and in females, although this difference was not significant after adjusting for possible confounding variables such as age. When the analyses were adjusted, no significant differences were shown in a-PWV according to the categories of MD adherence. Importantly, although a high adherence to the MD was associated with higher a-PWV values, the a-PWV values were not considered as pathological. At present, there is no standardized determination of what values are considered as pathological in relation to a-PWV, but different studies have indicated that a-PWV above 13 m/s is a strong predictor of cardiovascular mortality [22,23].

Previous evidence has shown a significant association between a high adherence to the MD and a reduction in cardiovascular mortality, as well as in the incidence of cardiovascular events, such as myocardial infarction and stroke [24], in the general population [25,26]. In particular, some studies have indicated that females may experience a greater reduction in CVD risk with a high adherence to the MD than males [7,27]. This dietary pattern, characterized by a high intake of fruits, vegetables, legumes, nuts, olive oil, and fish, together with a moderate intake of wine, seems to favor different cardiovascular parameters [28], including blood pressure, cholesterol levels, and inflammatory markers, thus contributing to better long-term cardiovascular health [29,30]. However, despite the growing evidence supporting the benefits of the MD on cardiovascular health, some studies have pointed to the possibility of research biases, such as variability in the definition and application of the MD in different populations and studies [31]. In addition, confounding factors such as socioeconomic status, lifestyle, and the genetics of participants may influence the results, suggesting that the observed benefits may not be exclusively attributable to the diet itself [32,33].

The association between arterial stiffness, considered as a subclinical marker of CVD, and the MD has been the subject of interest in recent studies [8,34,35]. Our findings in the unadjusted analyses did show a significant association between adherence to the MD and values of arterial stiffness, as assessed by a-PWV, possibly due to the consumption of animal products. Previous research has suggested that a vegetarian diet rich in fruits, vegetables, and legumes may be associated with a lower arterial stiffness than an omnivorous diet [36]. These beneficial effects of the vegetarian diet are attributed to its high content of antioxidants and fiber and low levels of saturated fat [37,38,39]. In addition, some studies have indicated that females following a vegetarian diet may experience a greater reduction in arterial stiffness than males, possibly due to hormonal and metabolic differences [40].

Although the MD, as a whole, is associated with improved cardiovascular health, some studies have explored the impact of various components of MD, such as olive oil, the daily consumption of vegetables and fruits, wine, fish, and nuts, on arterial stiffness [25,41]. Olive oil, which is rich in monounsaturated fatty acids and antioxidants, can improve endothelial function and reduce inflammation, thus reducing arterial stiffness [42]. The regular consumption of vegetables and fruits, which have high contents of fiber, vitamins, and antioxidants, is also associated with lower levels of arterial stiffness due to their beneficial effects on blood pressure and oxidative stress [43]. A moderate consumption of red wine, which contains polyphenols such as resveratrol, may improve vasodilation and reduce arterial stiffness [44]. Similarly, fish, a source of omega-3 fatty acids, contributes to arterial elasticity through its anti-inflammatory and lipid-profile-improving properties [45]. Finally, nuts, which are rich in healthy fats, fiber, and antioxidants, have been shown to reduce inflammation and improve vascular function [42]. Together, these MD whole foods act synergistically to improve arterial health, resulting in a reduced arterial stiffness in the general population [25,41].

Despite these benefits, our study showed that adherence to these foods is associated with increased arterial stiffness values, possibly due to confounding factors such as age, so their interpretation requires a careful analysis of confounders. As people age, they are more likely to adopt healthy dietary patterns, such as the MD, in an effort to improve their overall health and prevent chronic diseases [46,47]. However, arterial stiffness tends to increase naturally with age due to structural and functional changes in the arteries, such as a loss of elasticity and increased fibrosis [48]. This increase in arterial stiffness with age may mask or attenuate the cardiovascular benefits of the MD [49]. Therefore, the observation of an increased arterial stiffness in older individuals with a high adherence to the MD could be a confounding factor, with age acting as an intermediate variable influencing both dietary adherence and arterial health.

The cross-sectional nature of this study does not allow us to establish a causal relationship between adherence to the MD and a-PWV. Our findings indicated that some participants had higher a-PWV values at the time of assessment, but it cannot be determined whether these values were influenced by adherence to the MD, as other confounding factors, such as PP [16], BMI [14], sex [19], or education level [14], may also influence a-PWV. These variables may influence each other, modifying the effect of the association. According to one study, adherence to the MD is better in women, but is indirectly related to BMI. People with a high BMI reduced their BMI when their adherence to the MD increased [19]. Another study related blood pressure values, which interfere with the PP, to the MD and reported that a higher adherence to the MD is inversely associated with hypertension [16], which, in turn, is associated with arterial stiffness [50]. In addition, a low educational level was also associated with a higher a-PWv [51]. For these reasons, more research is needed in this field, including long-term studies and on different types of populations with large sample sizes, and other confounding factors should be considered to better elucidate our results.

In addition to the cross-sectional design, our study has other limitations that should be considered. First, although the sample size was acceptable, it is possible that a larger sample size could produce more statistically significant associations. Second, the EVasCu study included a specific Spanish population; therefore, it cannot be compared with other populations unless the sociodemographic characteristics are similar to those of our study population. Third, data on adherence to the MD were collected through a self-administered questionnaire, so the responses could be biased. Although our study provides evidence on the association between MD adherence and arterial stiffness in a healthy adult population, these limitations should be considered when interpreting the results.

## 5. Conclusions

Based on the results obtained in the EVasCu study, it can be concluded that, in the unadjusted analyses, healthy subjects with a high adherence to the MD showed higher levels of a-PWV, both in the total sample and in females, although significance was lost after adjusting for age. When the analyses were adjusted, no significant differences were shown in a-PWV according to the categories of MD adherence. Importantly, although a high MD adherence was associated with higher a-PWV values, the a-PWV values were not considered as pathological. These findings have clinical significance, since the identification and correction of preventable risk factors, such as arterial stiffness, play fundamental roles in the pathogenesis and progression of CVD. However, further studies are needed to elucidate the mechanisms underlying the association between MD adherence and arterial stiffness, considering possible confounding factors.

## Figures and Tables

**Table 1:** Characteristics of the study participants.

	Total (n = 386)	Male (n = 143)	Female (n = 243)	*p* Value
**Age (years)**	41.92 ± 13.14	42.33 ± 12.49	41.69 ± 13.53	0.644
**Educational level, n (%)**				0.870
Cannot read or write/no studies	0	0	0	
Primary education	4 (1.0)	1 (0.7)	3 (1.3)	
Secondary education/professional formation/higher education	160 (41.9)	59 (41.5)	101 (42.1)	
University degree	218 (57.1)	82 (57.7)	136 (56.7)	
Body mass index (kg/m^2^)	24.88 ± 4.25	25.66 ± 3.55	24.42 ± 4.56	0.005
Pulse pressure (mmHg)	46.23 ± 10.22	52.48 ± 9.79	42.52 ± 8.55	<0.001
**Arterial stiffness**				
Aortic pulse wave velocity (m/s)	6.34 ± 1.35	6.53 ± 1.34	6.22 ± 1.35	0.031
**Adherence to Mediterranean diet, n (%)**				0.632
Low adherence	106 (27.5)	42 (29.4)	64 (26.3)	
Medium adherence	141 (36.5)	48 (33.6)	93 (38.3)	
High adherence	139 (36.0)	53 (37.0)	86 (35.4)	

The data are shown as the mean ± standard deviation (SD) or number of subjects (percentage), n (%). *p* value = gender differences.

**Table 2:** Unadjusted and adjusted differences in arterial stiffness and adherence to the Mediterranean diet (low adherence, medium adherence, and high adherence) according to Student’s *t* test and ANCOVA for the total population and by sex.

	n	a-PWV, m/s (Mean ± SD)	Post Hoc	Unadjusted, *p* Value	Model 1, *p* Value	Model 2, *p* Value
**MEDAS 14**						
**Total**	386		Low adherence < High adherence	<0.001	0.843	0.695
Low adherence	106	5.91 ± 1.33				
Medium adherence	141	6.34 ± 1.38				
High adherence	139	6.63 ± 1.27				
**Male**	143		-	0.318	0.928	0.910
Low adherence	42	6.27 ± 1.34				
Medium adherence	48	6.58 ± 1.43				
High adherence	53	6.69 ± 1.25				
**Female**	243		Low adherence < High adherence	<0.001	0.831	0.566
Low adherence	64	5.72 ± 1.29				
Medium adherence	93	6.22 ± 1.32				
High adherence	86	6.60 ± 1.29				

a-PWV: Aortic pulse wave velocity; MEDAS: Mediterranean Diet Adherence Screener. Total: Model 1: Adjusted for age and sex; Model 2: Adjusted for age, sex, pulse pressure, body mass index, and educational level. In the sex stratification (male and female), Model 1 was adjusted for age, and Model 2 was adjusted for age, pulse pressure, body mass index, and educational level.

**Table 3:** Unadjusted and adjusted differences in arterial stiffness and adherence to the Mediterranean diet (adherence or nonadherence) using Student’s *t* test and ANCOVA according to the items of the Medas-14 questionnaire for the total population.

	n	a-PWv, m/s (Mean ± SD)	Unadjusted, *p* Value	Model 1, *p* Value	Model 2, *p* Value
**MEDAS 1 (olive oil)**	386		0.836	0.979	0.987
Adherence (yes)	367	6.34 ± 1.36			
No adherence (no)	19	6.27 ± 1.28			
**MEDAS 2 (olive oil)**	386		0.002	0.898	0.694
Adherence (≥4)	258	6.49 ± 1.35			
No adherence (<4)	128	6.03 ± 1.30			
**MEDAS 3 (vegetables)**	386		<0.001	0.756	0.918
Adherence (≥2)	264	6.49 ± 1.36			
No adherence (<2)	122	6.00 ± 1.28			
**MEDAS 4 (fruit)**	386		<0.001	0.248	0.285
Adherence (≥3)	181	6.64 ± 1.28			
No adherence (<3)	205	6.07 ± 1.36			
**MEDAS 5 (red and processed meat)**	386		0.290	0.964	0.564
Adherence (<1)	239	6.39 ± 1.34			
No adherence (≥1)	147	6.24 ± 1.38			
**MEDAS 6 (butter, margarine, cream)**	386		0.181	0.423	0.820
Adherence (<1)	257	6.27 ± 1.45			
No adherence (≥1)	129	6.27 ± 1.30			
**MEDAS 7 (sugar-sweetened beverages)**	386		0.403	0.786	0.635
Adherence (<1)	252	6.29 ± 1.35			
No adherence (≥1)	134	6.42 ± 1.36			
**MEDAS 8 (wine)**	386		<0.001	0.601	0.657
Adherence (≥7)	29	7.33 ± 1.33			
No adherence (<7)	357	6.26 ± 1.32			
**MEDAS 9 (pulses)**	386		0.499	0.302	0.516
Adherence (≥3)	186	6.29 ± 1.34			
No adherence (<3)	200	6.39 ± 1.37			
**MEDAS 10 (fish and seafood)**	386		0.007	0.171	0.184
Adherence (≥3)	205	6.51 ± 1.40			
No adherence (<3)	181	6.14 ± 1.27			
**MEDAS 11 (sweet and pastries)**	386		0.617	0.973	0.887
Adherence (<2)	222	6.37 ± 1.32			
No adherence (≥2)	164	6.30 ± 1.40			
**MEDAS 12 (nuts)**	386		0.015	0.651	0.933
Adherence (≥3)	251	6.46 ± 1.40			
No adherence (<3)	135	6.11 ± 1.23			
**MEDAS 13 (preference for white over red meat)**	386		0.391	0.839	0.350
Adherence (yes)	307	6.31 ± 1.35			
No adherence (no)	79	6.45 ± 1.39			
**MEDAS 14 (sofrito)**	396		0.797	0.051	0.010
Adherence (≥2)	312	6.33 ± 1.31			
No adherence (<2)	74	6.37 ± 1.52			

a-PWV: Aortic pulse wave velocity; MEDAS: Mediterranean Diet Adherence Screener. Model 1: Adjusted for age and sex; Model 2: Adjusted for age, sex, pulse pressure, body mass index, and education level.

**Table 4:** Unadjusted and adjusted differences in arterial stiffness and adherence to the Mediterranean diet (adherence or nonadherence) using Student’s *t* test and ANCOVA according to the items of the Medas-14 questionnaire for males.

	n	a-PWv, m/s (Mean ± SD)	Unadjusted, *p* Value	Model 1, *p* Value	Model 2, *p* Value
**MEDAS 1 (olive oil)**	143		0.738	0.256	0.462
Adherence (yes)	137	6.54 ± 1.35			
No adherence (no)	6	6.35 ± 1.29			
**MEDAS 2 (olive oil)**	143		0.277	0.769	0.608
Adherence (≥4)	84	6.64 ± 1.33			
No adherence (<4)	59	6.37 ± 1.35			
**MEDAS 3 (vegetables)**	143		0.237	0.783	0.817
Adherence (≥2)	88	6.64 ± 1.39			
No adherence (<2)	55	6.36 ± 1.26			
**MEDAS 4 (fruit)**	143		0.700	0.674	0.692
Adherence (≥3)	73	6.57 ± 1.29			
No adherence (<3)	70	6.49 ± 1.40			
**MEDAS 5 (red and processed meat)**	143		0.409	0.770	0.845
Adherence (<1)	86	6.61 ± 1.29			
No adherence (≥1)	57	6.42 ± 1.42			
**MEDAS 6 (butter, margarine, cream)**	143		0.437	0.901	0.531
Adherence (<1)	99	6.47 ± 1.25			
No adherence (≥1)	44	6.66 ± 1.53			
**MEDAS 7 (sugar-sweetened beverages)**	143		0.669	0.900	0.688
Adherence (<1)	97	6.50 ± 1.29			
No adherence (≥1)	46	6.60 ± 1.45			
**MEDAS 8 (wine)**	143		<0.001	0.293	0.443
Adherence (≥7)	18	7.57 ± 1.54			
No adherence (<7)	125	6.38 ± 1.25			
**MEDAS 9 (pulses)**	143		0.117	0.036	0.024
Adherence (≥3)	81	6.68 ± 1.46			
No adherence (<3)	62	6.33 ± 1.15			
**MEDAS 10 (fish and seafood)**	143		0.316	0.345	0.338
Adherence (≥3)	79	6.63 ± 1.38			
No adherence (<3)	64	6.41 ± 1.30			
**MEDAS 11 (sweet and pastries)**	143		0.894	0.357	0.278
Adherence (<2)	82	6.52 ± 1.26			
No adherence (≥2)	61	6.55 ± 1.45			
**MEDAS 12 (nuts)**	143		0.534	0.314	0.202
Adherence (≥3)	100	6.58 ± 1.36			
No adherence (<3)	43	6.42 ± 1.31			
**MEDAS 13 (preference for white over red meat)**	143		0.109	0.183	0.075
Adherence (yes)	105	6.42 ± 1.28			
No adherence (no)	38	6.83 ± 1.48			
**MEDAS 14 (sofrito)**	143		0.208	0.929	0.769
Adherence (≥2)	117	6.46 ± 1.32			
No adherence (<2)	26	6.83 ± 1.41			

a-PWV: Aortic pulse wave velocity; MEDAS: Mediterranean Diet Adherence Screener. Model 1: Adjusted for age; Model 2: Adjusted for age, pulse pressure, body mass index, and educational level.

**Table 5:** Unadjusted and adjusted differences in arterial stiffness and adherence to the Mediterranean diet (adherence and nonadherence) using Student’s *t* test and ANCOVA according to the items of the Medas-14 questionnaire for females.

	n	a-PWv, m/s (Mean ± SD)	Unadjusted, *p* Value	Model 1, *p* Value	Model 2, *p* Value
**MEDAS 1 (olive oil)**	243		0.965	0.457	0.800
Adherence (yes)	230	6.22 ± 1.35			
No adherence (no)	13	6.24 ± 1.33			
**MEDAS 2 (olive oil)**	243		<0.001	0.953	0.812
Adherence (≥4)	174	6.41 ± 1.36			
No adherence (<4)	69	5.74 ± 1.20			
**MEDAS 3 (vegetables)**	243		<0.001	0.460	0.966
Adherence (≥2)	176	6.42 ± 1.35			
No adherence (<2)	67	5.70 ± 1.22			
**MEDAS 4 (fruit)**	243		<0.001	0.411	0.568
Adherence (≥3)	108	6.69 ± 1.28			
No adherence (<3)	135	5.85 ± 1.29			
**MEDAS 5 (red and processed meat)**	243		0.436	0.778	0.480
Adherence (<1)	153	6.28 ± 1.35			
No adherence (≥1)	90	6.13 ± 1.35			
**MEDAS 6 (butter, margarine, cream)**	243		0.226	0.295	0.331
Adherence (<1)	158	6.15 ± 1.32			
No adherence (≥1)	85	6.37 ± 1.40			
**MEDAS 7 (sugar-sweetened beverages)**	243		0.401	0.823	0.986
Adherence (<1)	155	6.17 ± 1.38			
No adherence (≥1)	88	6.32 ± 1.30			
**MEDAS 8 (wine)**	243		0.073	0.458	0.528
Adherence (≥7)	11	6.94 ± 0.79			
No adherence (<7)	232	6.19 ± 1.36			
**MEDAS 9 (pulses)**	243		0.492	0.720	0.190
Adherence (≥3)	105	6.15 ± 1.25			
No adherence (<3)	138	6.28 ± 1.42			
**MEDAS 10 (fish and seafood)**	243		0.012	0.274	0.267
Adherence (≥3)	126	6.43 ± 1.42			
No adherence (<3)	117	6.00 ± 1.24			
**MEDAS 11 (sweet and pastries)**	243		0.894	0.357	0.278
Adherence (<2)	103	6.28 ± 1.35			
No adherence (≥2)	140	6.15 ± 1.36			
**MEDAS 12 (nuts)**	243		0.018	0.829	0.329
Adherence (≥3)	151	6.38 ± 1.43			
No adherence (<3)	92	5.96 ± 1.17			
**MEDAS 13 (preference for white over red meat)**	243		0.541	0.310	0.493
Adherence (yes)	202	6.25 ± 1.38			
No adherence (no)	41	6.11 ± 1.20			
**MEDAS 14 (sofrito)**	243		0.577	0.034	0.004
Adherence (≥2)	195	6.25 ± 1.31			
No adherence (<2)	48	6.13 ± 1.53			

a-PWV: Aortic pulse wave velocity; MEDAS: Mediterranean Diet Adherence Screener. Model 1: Adjusted for age; Model 2: Adjusted for age, pulse pressure, body mass index, and educational level.

## Data Availability

The original contributions presented in the study are included in the article, further inquiries can be directed to the corresponding author.
